# Slip and tilt: modeling falls over railings

**DOI:** 10.1007/s00414-020-02432-8

**Published:** 2020-10-09

**Authors:** H. Muggenthaler, M. Hubig, A. Meierhofer, G. Mall

**Affiliations:** 1 Institute of Legal Medicine, Jena University Hospital, Friedrich Schiller University Jena, Jena, Germany; 2 Institute of Legal Medicine, Medical University of Graz, Graz, Austria

**Keywords:** Fall analysis, Moment equilibrium, Force equilibrium, Anti-slip condition, Anti-tilt condition

## Abstract

Falls over railings are frequent case scenarios forensic experts are confronted with. An important issue is the differentiation of accidental and non-accidental falling scenarios. From a biomechanical point of view, this is a challenging task and should be addressed in a multifactorial approach. This work presents a simplified mechanical model in terms of a cranked rod that can be used in cases without relevant dynamic components in terms of pushing or jumping. If the anti-slip and the anti-tilt condition are violated, the possibility for a person to get over a railing should be assumed and investigated in more detail. Because our approach also involves uncertainties, the formulae should be understood to be part of a multifactorial approach. Numerical simulation, experimental reconstruction, injury pattern, and trace analysis can yield additional substantial connecting facts.

## Introduction

Falls over railings are frequent case scenarios forensic experts are confronted with. Issues like injury severity [1, 2], which depends on falling height and landing substrate, as well as the differentiation of accidental and non-accidental causes of falls [3, 4] have to be addressed. When a victim is being pushed against a railing or when a person actively jumps over or from a balustrade, the landing point can show some horizontal distance from the balustrade [5]. A distinct horizontal distance between the railing and the landing point can be an important indicator for non-accidental falls. Especially in cases where a victim got over a railing without relevant initial horizontal velocity, it is of utmost interest whether, for example, leaning against a railing can result in accidental overcoming the safeguard or not.

The methods presented in the following were motivated by a real case with a runty woman (1.58 m, 65 kg) that fell over a balcony railing. The case circumstances led to the assumption of a non-accidental scenario. However, the woman deposed that while she would have hung out the laundry on the balcony, a tablecloth would have slipped over the handrail of the balcony. As she would have tried to catch the slipping tablecloth, she would have lost balance and would have fallen from the third floor’s balcony. The falling height was about 8 m. She sustained fractures of the right metacarpus (ossa metacarpalia 3, 4, and 5), of the first and fourth lumbar spinal body and of the os sacrum. The railing height was 0.92 m.

In [6] a detailed reconstruction of two falling cases is presented. The author proposes a kind of falling condition as follows: “A fall from a balcony can occur if a person leans over the balcony and overbalances.” This wording allows different mechanical interpretations and does not represent a scientific mechanical condition. For example, one could state that a fall occurs when the person’s center of mass moves over the handrail of a railing. However, this definition disregards friction forces that counteract rotations over railings.

Even though, considering only quasi-static scenarios without relevant dynamic components (e.g., pushing), conventional engineering mechanics approaches prove to be of high complexity. This may explain the lack of literature dealing with such issues, especially in the forensic context.

Simulation approaches were published dealing with the fall kinematics [7] or with cases where the circumstances suggested pushing against the railing [3]. Quasi-static scenarios with falls over railings cannot be modeled easily applying passive human models.

We present a technical mechanics based approach that incorporates both inertial forces and friction forces. By defining two conditions called *slip* and *tilt* or *anti-slip* and *anti-tilt*, we developed formulae that can be used for the assessment of body positions that theoretically can lead or cannot lead to a fall over a railing.

The main objective of this work was to show the complexity of falling scenarios and to provide a scientific-based method that can support the forensic expert in real case work. Applying the proposed formulae, the forensic expert should be able to predict whether realistic body positions exist that can result in a fall over a railing.

## Materials and methods

**Fig. 1:**
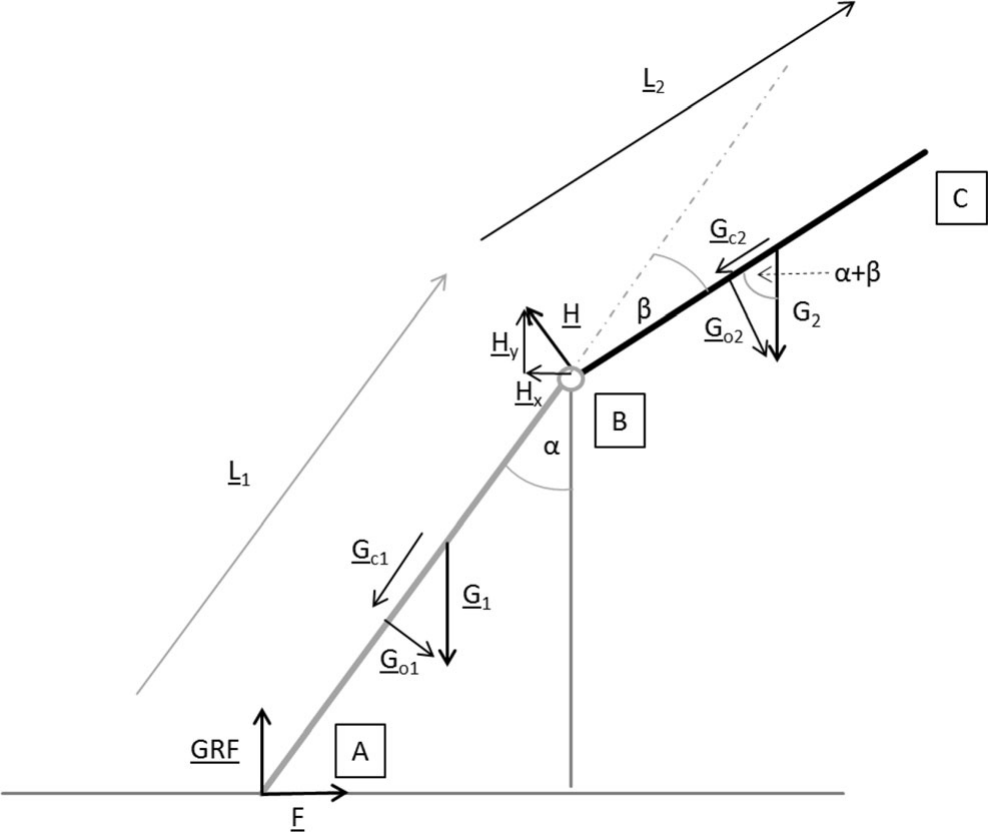
Simple mechanical rod model, representing human body leaning against railing

In terms of a simple mechanical model, a human body leaning against a railing can be considered a cranked rod (see Fig. 1). At point A, the model contacts the ground corresponding to the persons’ feet-ground contact. Point B represents the pivot point where the body rotates over the railing, and point C is the most distal body region (parietal). L_1_ represents the length of the body part below, and L_2_ represents the length of the body part above the center of rotation.

We use the usual nomenclature for two-dimensional vector spaces: The underlined variables are two-dimensional vectors. Moments are cross products of type lever x force where the *x* means:

$$ \left(\begin{array}{c}x\\ {}y\end{array}\right)\times \left(\begin{array}{c}u\\ {}v\end{array}\right):= xv- yu $$

The following variables need to be defined:

- *L*_*1*_, *L*_*2*_: Length of body parts below/above center of rotation B
- *a*, *b*: Scaling factors for *L*_*1*_, *L*_*2*_ to get COG location (not shown in Fig. 1): *a∙L*_*1*_ = arrow connecting A with COG_1_ and *b∙L*_*2*_ = arrow connecting B with COG_2_
- *G*_*1*_: Weight force of body part below center of rotation B
- *G*_*2*_: Weight force of body part above center of rotation B
- *G*_*o1*_: L_1_-orthogonal component of G_1_
- *G*_*c1*_: L_1_-collinear component of G_1_
- *G*_*o2*_: L_2_-orthogonal component of G_2_
- *G*_*c2*_: L_2_-collinear component of G_2_
- *H*: Bearing force at the center of rotation B
- *GRF*: Ground reaction force at point A
- *F*: Friction force at point A
- *α*: Angle between railing and lower body
- *β*: Angle between lower and upper body

**Fig. 2:**
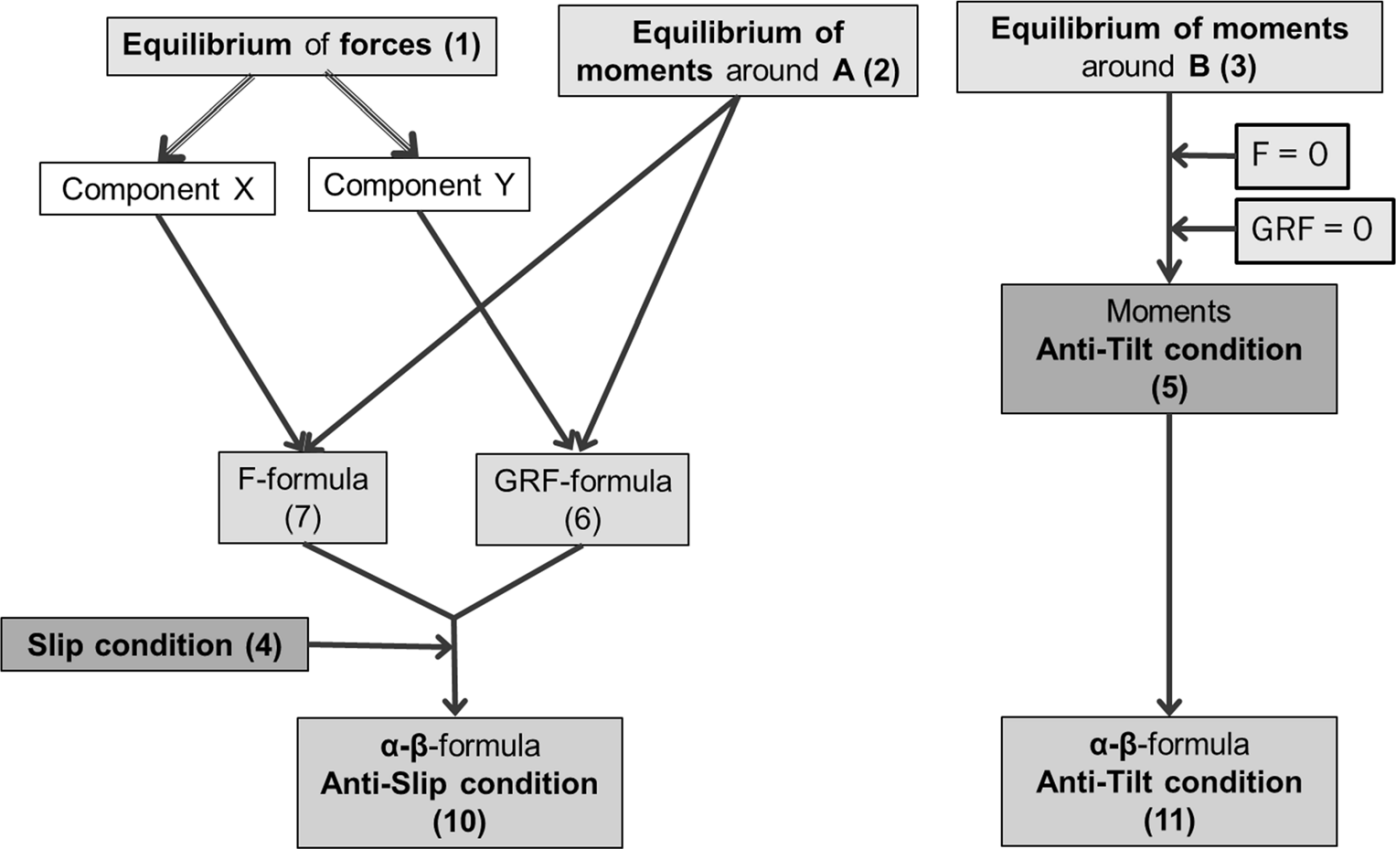
Structure of the mathematical argumentation leading to the α-β-anti-slip and anti-tilt-condition

As a common approach in engineering mechanics, equilibriums of forces and moments form a system of linear equations with known and unknown variables. The following chart in Fig. 2 shows the derivation’s structure for our final results, the α-β anti-slip and anti-tilt Formulae (9) and (10), respectively.

Equilibrium of forces:

1 $$ \underset{\_}{G_1}+{\underset{\_}{G}}_2+\underset{\_}{GRF}+\underset{\_}{F}+\underset{\_}{H}=0 $$

Equilibrium of moments with respect to point A:

2 $$ \underset{\_}{H}\times \underset{\_}{L_1}+\underset{\_}{G_1}\times a\cdot \underset{\_}{L_1}+\underset{\_}{G_2}\times \left(\underset{\_}{L_1}+b\cdot \underset{\_}{L_2}\right)=0 $$

Equilibrium of moments with respect to point B:

3 $$ \left(\underset{\_}{GRF}+\underset{\_}{F}\right)\times \left(-\underset{\_}{L_1}\right)+\underset{\_}{G_1}\times \left(-\left(1-a\right)\right)\cdot \underset{\_}{L_1}+\underset{\_}{G_2}\times b\cdot \underset{\_}{L_2}=0 $$

Formulae (1), (2), and (3) form a system of equations which are linear in the forces and in the levers.

We now can define the following two conditions:

The anti-slip condition is fulfilled for friction forces *F* lower than the ground reaction force *GRF* multiplied by the friction coefficient *μ*.

4 $$ F\le \mu \cdotp GRF $$

The anti-tilt condition is fulfilled if the left net moment is greater than the right net moment with respect to the pivot point B. Imagine the very beginning of a tilt motion with feet losing ground contact to determine the upper threshold of the anti-tilt interval. Then, Formula (3) can be evaluated without taking the moments caused by the friction force F and the ground reaction force *GRF* into account. Setting *F = 0* and GRF = 0, we get the following anti-tilt condition:

5 $$ -\underset{\_}{G_1}\times \left(1-a\right)\cdot \underset{\_}{L_1}\ge -\underset{\_}{G_2}\times b\cdot \underset{\_}{L_2} $$

Using Formulae (1), (2), and (3) with the trigonometric vector components derived from Fig. 1, we get expressions for the absolute values *GRF* and *F* of ground reaction force and of friction force, respectively.

6 $$ GRF={G}_1\left(1-a\cdot {\sin}^2\alpha \right)+{G}_2\cdot \left({\cos}^2\alpha -b\cdot \frac{L_2}{L_1}\cdot \sin \alpha \cdot \sin \left(\alpha +\beta \right)\right) $$

7 $$ F=\left(a\cdotp {G}_1\cdotp \mathit{\sin}\alpha +{G}_2\cdotp \mathit{\sin}\alpha +b\cdotp {G}_2\cdotp \frac{L_2}{L_1}\cdotp \mathit{\sin}\left(\alpha +\beta \right)\right)\cdotp \mathit{\cos}\alpha $$

Inserting (6) and (7) into (4) gives the following anti-slip expression:

8 $$ \left(a\cdot {G}_1\cdot \sin \alpha +{G}_2\cdot \sin \alpha +b\cdot {G}_2\cdot \frac{L_2}{L_1}\cdot \sin \left(\alpha +\beta \right)\right)\cdot \cos \alpha \le \mu \cdot \left({G}_1\left(1-a\cdot {\sin}^2\alpha \right)+{G}_2\cdot \left({\cos}^2\alpha -b\cdot \frac{L_2}{L_1}\cdot \sin \alpha \cdot \sin \left(\alpha +\beta \right)\right)\right) $$

Solving (8) for *β* yields the final *anti-slip condition*:

9 $$ \beta \le {\sin}^{-1}\left[\frac{\mu }{b}\cdot \frac{G_1}{G_2}\cdot \frac{L_1}{L_2}\cdot \frac{\sin \alpha \cdot \cos \alpha }{\cos \alpha +\mu \cdot \sin \alpha}\cdot \left(\frac{1}{\sin \alpha \cdot \cos \alpha }-a\cdot \tan \alpha +\frac{G_2}{G_1}\cdot \cot \alpha -\frac{1}{\mu}\cdot \left(a+\frac{G_2}{G_1}\right)\right)\right] $$

Reversing the “≤” sign to a “>” sign in (9) leads to the complementary *slip condition*.

Inserting (6) and (7) into (5) gives the following expression representing the *anti-tilt condition*:

10 $$ \beta \le {\sin}^{-1}\left(\frac{L_1\cdotp a\cdotp {G}_1+{L}_1\cdotp {G}_2}{G_2\cdotp b\cdotp {L}_2}\cdotp \sin \alpha \right)-\alpha $$

Again we reach the complementary *tilt condition* by substituting the “≤” sign by a “>” sign in (10).

A fall over the railing can occur if both the anti-slip condition and the anti-tilt condition are violated or conversely if the slip condition and the tilt condition are fulfilled simultaneously.

Maple 2016 was applied for the solution of Formulae (9) and (10). It has to be considered that the *arcsin* function (*sin*^*−1*^) has two equivalent solutions. Formula (10), for example, can be written as follows:

11 $$ {\displaystyle \begin{array}{l}\begin{array}{l}\mathit{\sin}\left({\beta}_1+\alpha \right)=\left(\frac{L_1\cdotp a\cdotp {G}_1+{L}_1\cdotp {G}_2}{G_2\cdotp b\cdotp {L}_2}\cdotp \sin \alpha \right)=\mathit{\sin}\left({\beta}_2+\alpha \right)\\ {}\mathit{\sin}\left({\beta}_1+\alpha \right)=\mathit{\sin}\left({\beta}_2+\alpha \right)\end{array}\\ {}{\beta}_2+\alpha =\pi -\left({\beta}_1+\alpha \right)\\ {}{\beta}_2=\pi -{\beta}_1-2\alpha \end{array}} $$

Formula (11) yields the second solution for both Formulae (9) and (10).

Since in real cases the overall body length L = L_1_ + L_2_ and the balustrades height Y are known, we can express L_1_ and L_2_ as functions of L, Y, and the angle α:

12 $$ {L}_1=\frac{Y}{cos\alpha} $$

13 $$ {L}_2=L-\frac{Y}{cos\alpha} $$

## Results

Referring to the case parameter in the introduction, a body length of 1.58 m and a body mass of m = 65 kg were used for the calculation of the results shown in the following. In our model, a homogeneous mass distribution of the cranked rod was assumed with scaling factors *a = b = 0.5*.

**Fig. 3:**
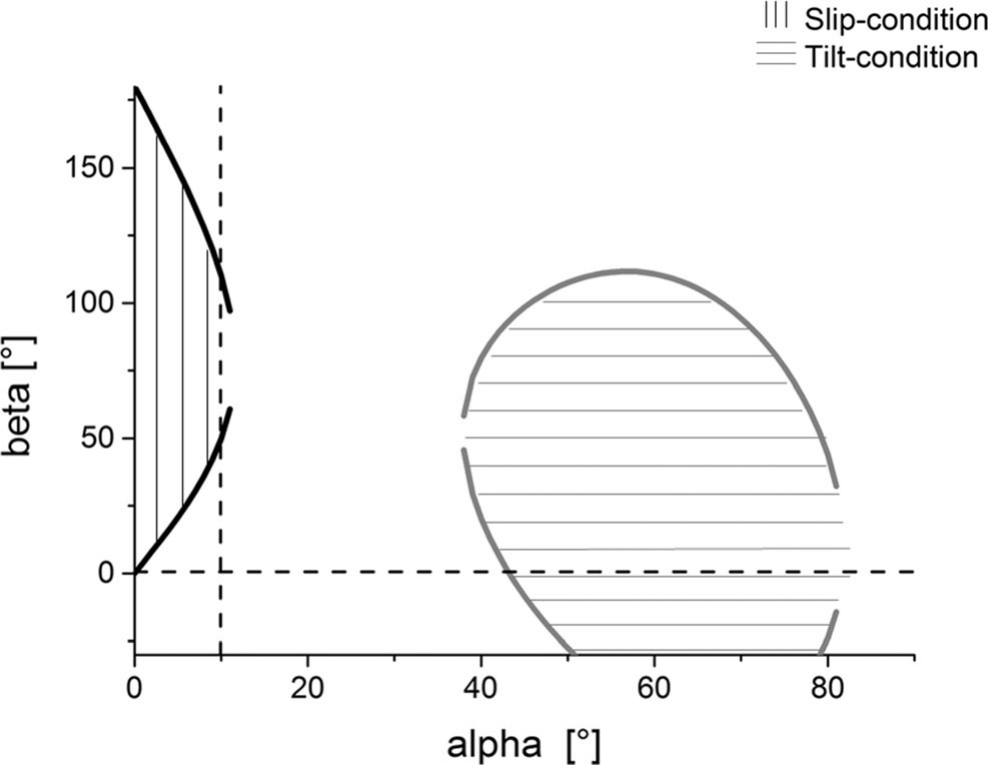
Illustrations of slip and tilt conditions for a friction coefficient of *μ* = 0.7

Figure 3 shows the instability conditions for a friction coefficient of *μ* = 0.7. Static friction coefficients between 0.5 and 0.7 are common for concrete-shoe contacts [8]. For example, at an angle of *α =* 10°, the slip condition is fulfilled for β between 50° and 110°, but the tilt condition is not fulfilled. Thus, there is no overlapping of shaded regions with slip and tilt condition. Thus, no α-β combination exists which simultaneously fulfills both the slip and tilt condition.

**Fig. 4:**
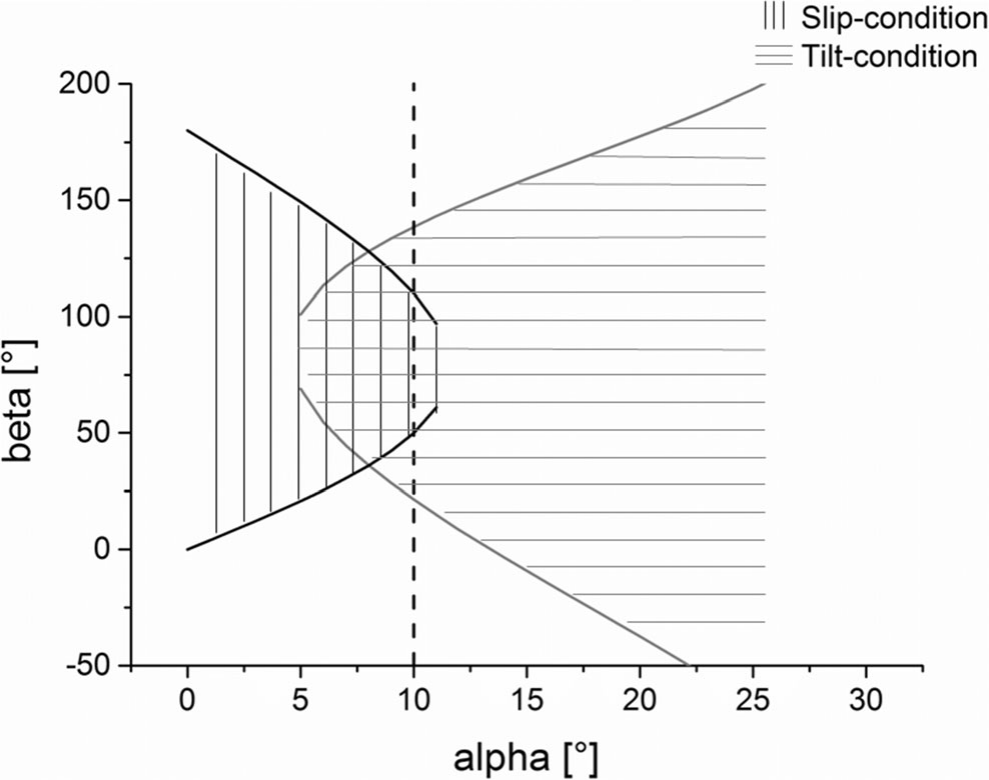
Illustrations of slip and tilt conditions for a friction coefficient of *μ* = 0.2

Applying a friction coefficient of *μ = 0.2*, we find overlapping instability conditions as illustrated in Fig. 4. For example, at an angle of *α* = 10°, a fall over the railing would theoretically be possible for angles of *β* of at least *β =* 50° and *β =* 110° at most.

**Fig. 5:**
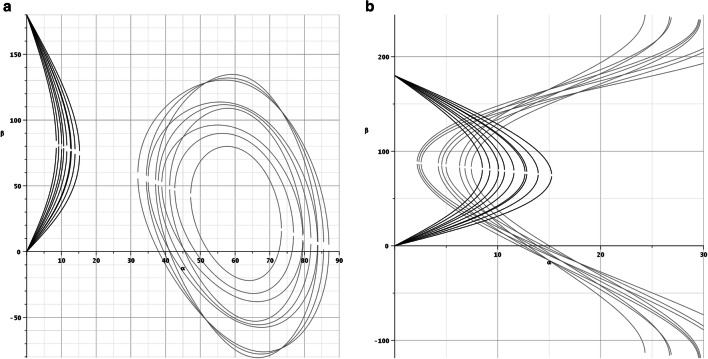
Sensitivity analysis for parameters a, *b* = 0.4, 0.5, and 0.6 with *μ* = 0.7 (left) and *μ* = 0.2 (right)

Figure 5 shows a sensitivity analysis result for the COG shift parameters a and b. The topology of the tilt and slip regions as well as of the overlap region for *μ* = 0.2 seems to be invariant under variation of a and b.

## Discussion

The aim of this work was to present a method for the assessment of potential accidental falls over railings. This appeared to be a very challenging task, because there are a lot of unknown parameters that have to be considered like active movements, friction forces, etc. Our approach therefore represents only one option for the assessments of falls in cases without relevant dynamic components in terms of pushing or jumping. Other objective clues can be obtained from the injury pattern [3] as well as from daktyloscopic [6] or moleculobiological traces. In order to resolve contradictory witness statements, also dynamic biomechanical reconstructions can be performed [5, 9, 10].

The formulae presented here require plausible input parameters especially in terms of the angle the person is leaning against a balustrade. Forensic experts need to check the input parameters for plausibility, e.g., by applying simple trigonometric functions.

**Fig. 6:**
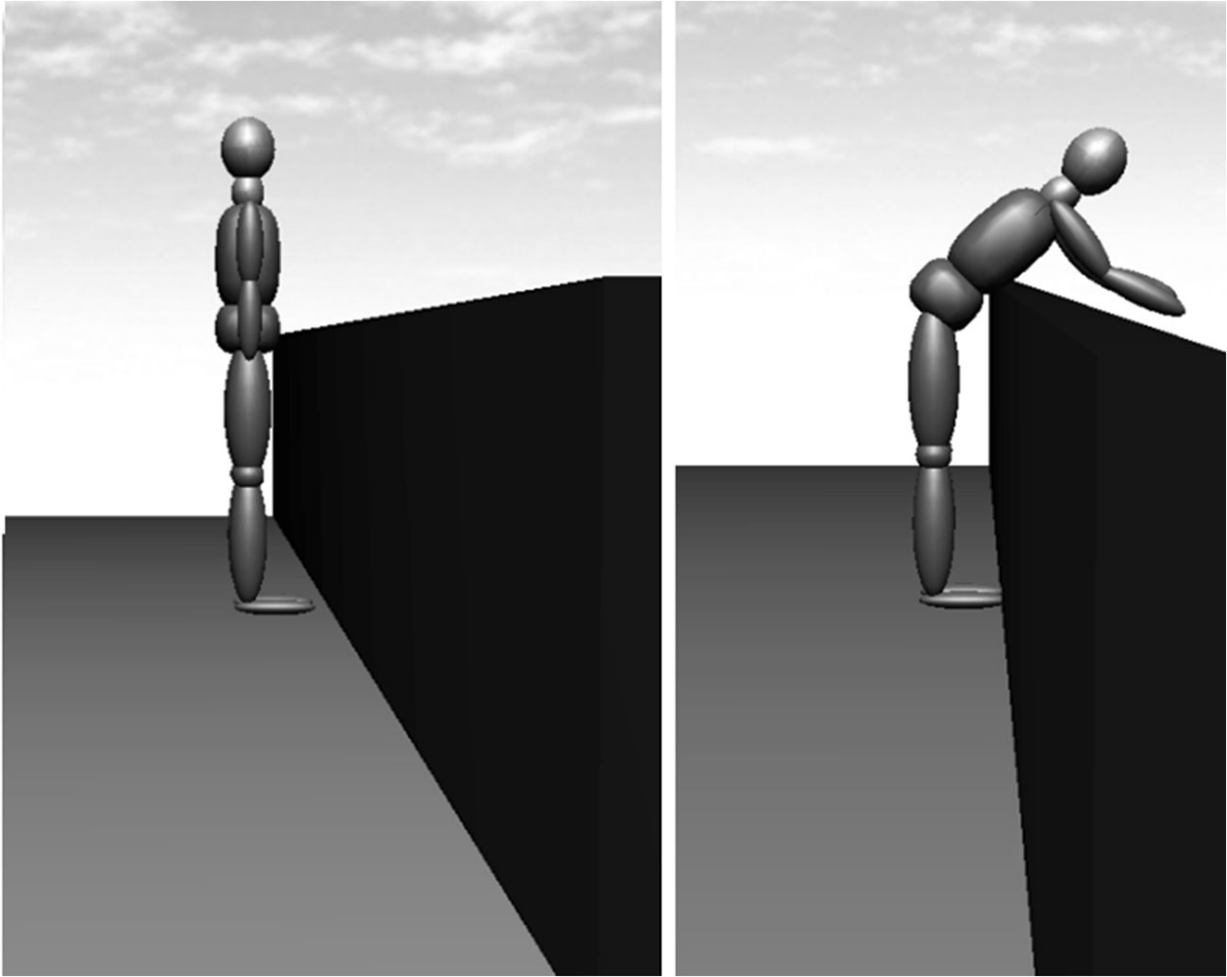
Scenario with an angle of *α* = 0° and rotation around the lower abdominal region

Figure 6 shows a scenario with the boundary conditions of our example case. For small angles *α*, the point of rotation corresponds to the lower abdominal and pelvic region, so that a forward flexion of the upper body can be accomplished. A flexion of the upper body pivoting around the handrail of the balustrade while the feet have contact to the ground can be regarded as a realistic scenario. The formulae for the anti-slip and anti-tilt conditions should yield plausible values.

**Fig. 7:**
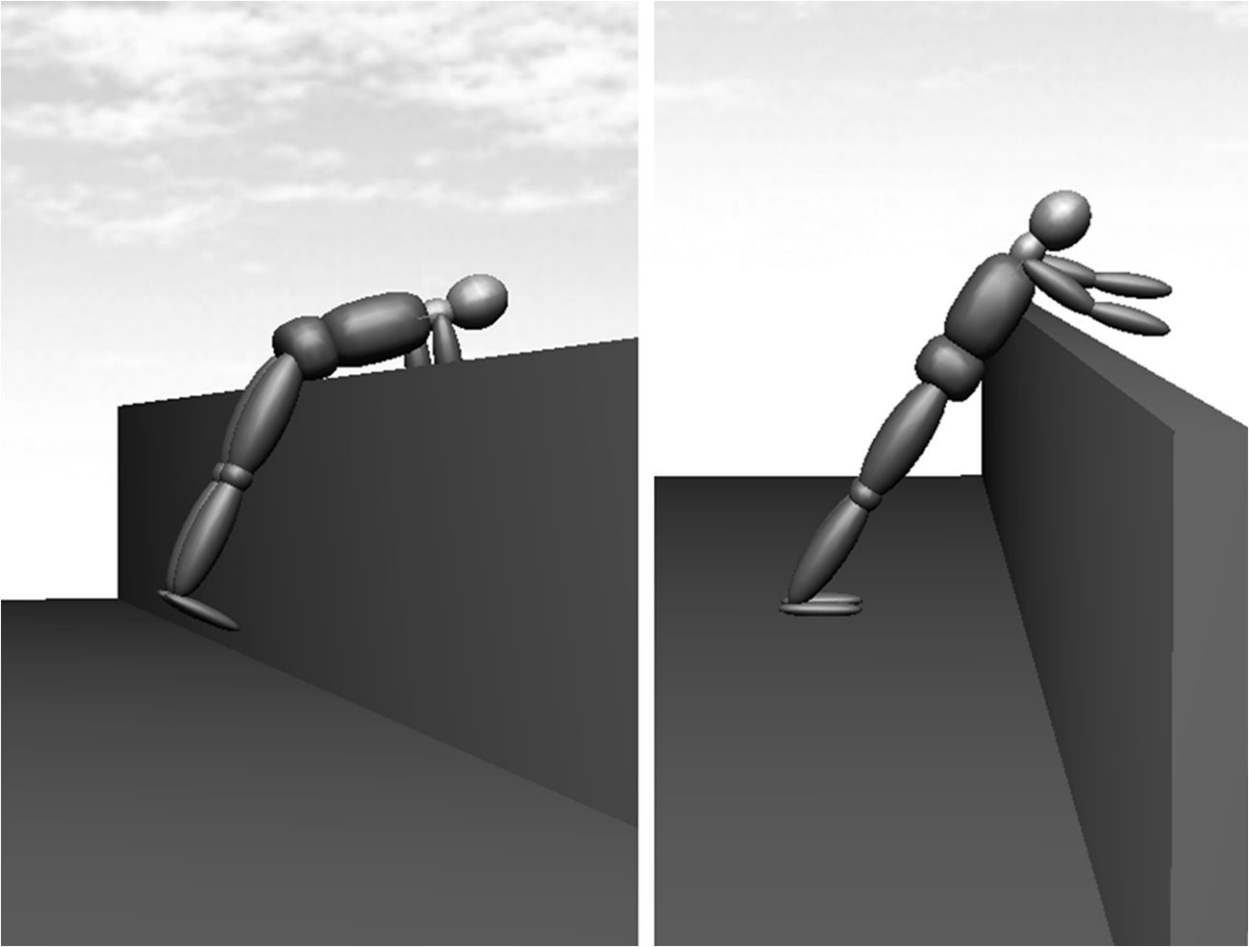
Scenario with an angle of *α* = 35°, without feet-ground contact (left) and point of rotation in the thoracic region (right)

Figure 7 shows a scenario with an angle of *α* = 35°. Such an angle *α* can only be realized when the feet lose contact to the ground or when the thoracic region contacts the handrail of the balcony. In both scenarios, the formulae presented cannot be applied. Firstly, in the left scenario of Fig. 7, the anti-slip condition is always violated, so that only the anti-tilt condition is applicable. Secondly, in the right scenario of Fig. 7, the point of rotation does not correspond to a joint, so that a forward flexion of the upper body around the pivot point with angles of *β >* 0° cannot be realized. The only possibility would be to use *β* = 0°. As the examples in Fig. 7 show, it is very important to check the matching of our simplified geometrical model to real-world anthropometric and railing geometry.

The calculations presented are based on a very simplified mechanical model in terms of a cranked rod. The following limitations have to be taken into account when applying the formulae developed above:

- In forensic case work, common questions concern the potential involvement of third parties in terms of pushing, beating, etc. [3]. Our model is a passive quasi-static model that cannot be used in cases with dynamic components like pushing, jumping, etc.
- Soft tissue deformation and friction forces at the point of rotation (point B in Fig. 1) are not considered in the model presented.
- Active movements, e. g., to prevent a fall over a railing, cannot be modeled in our approach.

Particularly the last item seems to be of utmost relevance. Persons loosing balance normally react to regain a stable body posture. Experimental tests dealing with that issue exist for falls without considering railings [11]. However, recovery strategies can vary between individuals [12], and the ability of fall preventive responses declines with higher ages [13].

Numerical simulations may have the potential to overcome uncertainties when assessing complex falling scenarios based on classical technical mechanics. While multibody models are capable of reproducing passive human kinematics, in general these models exhibit shortcomings in contact characteristics and quantitative load analysis. The kinematics in falling scenarios has been investigated based on multibody dynamics for example in [7, 14]. Also, first efforts have been made in the application of active multibody models [15] based on experimental tests [11]. Using finite element simulation, soft tissue deformations and material failure can be modeled [16, 17]. However, up to now, there is no general purpose FE model available that can be routinely employed by forensic experts in simulation-based reconstructions of tilt and slip falling scenarios.

## Conclusion

The reconstructions of potential accidental falls represent a challenging task. This work presents a simplified mechanical model that can be used in cases without relevant dynamic components in terms of pushing or jumping.

The key messages of the current work are:

- Applying our model in practical casework mainly investigates the “null hypothesis” which usually states that the fall occurred because of losing “balance.”
- Our approach involves uncertainties too. These uncertainties need to be discussed when using the formulae presented at court.
- The formulae should be understood to be part of a multifactorial approach. Numerical simulation, experimental reconstruction, and trace analysis can yield additional substantial connecting facts.
